# Paradoxical GH increase after oral glucose load in subjects with and without acromegaly

**DOI:** 10.1007/s40618-023-02138-9

**Published:** 2023-06-21

**Authors:** F. Ceccato, C. K. Vedolin, G. Voltan, G. Antonelli, M. Barbot, D. Basso, D. Regazzo, C. Scaroni, G. Occhi

**Affiliations:** 1 Department of Medicine DIMED, University of Padova, Padua, Italy; 2 Endocrine Disease Unit, University-Hospital of Padova, Padua, Italy; 3 Laboratory Medicine, University-Hospital of Padova, Padua, Italy; 4 Department of Biology, University of Padova, Via Via U. Bassi 58B, 35121 Padua, Italy

**Keywords:** Acromegaly, IGF-1, Paradoxical response, Oral glucose tolerance test, GIP

## Abstract

**Objective:**

A paradoxical GH rise after the glucose load (GH-Par) is described in about one-third of acromegalic patients. Here, we evaluated the GH profile in subjects with and without acromegaly aiming to refine the definition of GH-Par.

**Design:**

Observational case–control study.

**Methods:**

Our cohort consisted of 60 acromegalic patients, and two groups of subjects presenting suppressed GH (< 0.4 µg/L) and high (non-acro^↑IGF−1^, n = 116) or normal IGF-1 levels (non-acro, n = 55). The distribution of GH peaks ≥ 120% from baseline, insulin, and glucose levels were evaluated over a 180-min time interval after glucose intake.

**Results:**

A similar proportion of subjects in all three groups shows a GH ratio of ≥ 120% starting from 120 min. Re-considering the definition of paradoxical increase of GH within 90 min, we observed that the prevalence of GH peaks ≥ 120% was higher in acromegaly than in non-acro^↑IGF−1^ and non-acro (respectively 42%, 16%, and 7%, both p < 0.001). In patients without GH-Par, a late GH rebound was observed in the second part of the curve. Higher glucose peak (p = 0.038), slower decline after load, 20% higher glucose exposure (p = 0.015), and a higher prevalence of diabetes (p = 0.003) characterized acromegalic patients with GH-Par (with respect to those without).

**Conclusions:**

GH-Par response may be defined as a 20% increase in the first 90 min after glucose challenge. GH-Par, common in acromegaly and associated with an increased prevalence of glucose metabolism abnormalities, is found also in a subset of non-acromegalic subjects with high IGF-1 levels, suggesting its possible involvement in the early phase of the disease.

## Introduction

Acromegaly is a chronic multisystemic disease, secondary in most cases to a GH-secreting pituitary adenoma [1]. Because of the slow progression of the symptoms over many years, clinical diagnosis of acromegaly is frequently delayed and patients often present systemic complications with increased morbidity and mortality [2].

In patients with a clinical suspicion of acromegaly and elevated IGF-1 levels, the investigation of GH suppression during an oral glucose tolerance test (OGTT) is recommended as a confirmatory test [3]. Indeed, the lack of GH inhibition is the hallmark of its autonomous secretion [4], although in patients with acromegaly GH levels may partially drop, rise paradoxically, or remain unchanged after an oral glucose challenge [5].

Besides its significance in the diagnostic flow-chart of acromegaly, the role of OGTT as a prognostic factor is increasingly being recognized in most recent years. In a study conducted on nearly 500 acromegalic patients, and subsequently confirmed by other independent cohorts [6–8], we observed that the patients with a paradoxical increase of GH after a glucose load (GH-Par, about one-third of cases) have a milder clinical phenotype compared to those lacking such a response (GH-NPar) [9]. The formers, indeed, are typically diagnosed at an older age and present smaller and less invasive pituitary lesions. Moreover, we showed that GH-Par patients respond better to first-generation somatostatin receptor ligands (SRL), thus suggesting that the GH secretion profile at OGTT may reflect some relevant biological features of GH-secreting pituitary adenomas [6, 7, 9, 10]. Among these features, the aberrant expression of the glucose-dependent insulinotropic polypeptide receptor (GIPR) is found in about 80% of GH-Par [11], and associated with the inappropriate activation of the GIP/GIPR axis: it was firstly proposed by Umahara and collegues [12], and then confirmed by our group [11, 13].

Despite the increasing importance of OGTT as a prognostic biomarker in acromegaly, the criteria to define the paradoxical response have not been uniquely defined [14], and the molecular bases underlying this phenomenon are not entirely clarified. Different studies set distinct cut-offs and intervals to define GH response as paradoxical [14]. These vary from an undefined “early GH rise” to increases ranging from 20 to 100% in GH levels up to 120 min after a glucose challenge [14]. On the other side, the paradoxical increase of GH after OGTT is not an exclusive feature of acromegaly [15]. It has indeed been reported in both normal (i.e., puberty) and pathological conditions (e.g., impaired glucose tolerance, anorexia nervosa, renal or liver failure) [15]. The reasons underlying this unexpected response of GH to a glucose load in these conditions are even less clear.

In this study, we focused on three main objectives. Firstly, we aimed to refine the parameters to better define the paradoxical response of GH to OGTT. Secondly, we intended to determine the prevalence of GH-Par in a series of subjects without acromegaly but with elevated IGF-1 levels. Thirdly, we sought to analyze the glucose and insulin curves after OGTT in both acromegalic and non-acromegalic patients based on their GH profiles.

## Materials and methods

### Study cohort

In this retrospective case–control study, we first conducted a dedicated query to search for GH curves after OGTT in the local electronic Case-Report Form. We initially identified 489 tests performed at the Endocrine Unit of Padova Hospital from January 2010 to September 2020. As a second step, we have filtered out all those records (patients) with an incomplete medical history or that fell under the exclusion criteria—i.e., all those conditions influencing the GH/IGF-1 axis, such as pregnancy, hepatic or renal failure, chronic inflammation, malnutrition, use of oral estrogen or glucocorticoid therapy. OGTT performed after pituitary surgery, as well as during patient’s follow-up, were excluded. The final cohort consisted of 231 tests and included subjects suspected of acromegaly—based on either physical signs (i.e., brow prominence, prognathism, macroglossia, acral overgrowth, lips, and nose enlargement), evidence of at least one increased IGF-1 levels, pituitary adenoma and signs/symptoms of acromegaly. According to GH suppression after OGTT and IGF-1 levels, subjects were then divided into three groups:

- New diagnosis of acromegaly (acro, n = 60): patients with IGF-1 levels above the upper limit of normality (ULN), and a GH^nadir^ ≥ 0.4 µg/L during OGTT. All acromegalic patients within this group were studied at baseline, during the initial diagnostic assessment of GH excess and before any treatment; a pituitary magnetic resonance imaging (MRI) study was performed in all subjects. Most of the patients belonging to this group have been previously reported [9, 16].
- Non-acromegalic with high IGF-1 levels (non-acro^↑IGF−1^, n = 116): patients with suspected acromegaly who presented with persistent increased IGF-1 levels and a GH^nadir^ < 0.4 µg/L during OGTT.
- Non-acromegalic (non-acro, n = 55): subjects with normal IGF-1 levels and a GH^nadir^ < 0.4 µg/L during OGTT. These subjects presented normal kidney and liver function, and were not taking any medication that can interfere with GH or IGF-1 secretion/activity.

Glucose metabolism alterations (GMAs) were defined according to the international criteria in impaired fasting glucose (IFG: basal glucose 5.5–7.0 mmol/L), impaired glucose tolerance (IGT: glucose levels 120 min after OGTT 7.8–11.1 mmol/L), and diabetes mellitus (DM: basal glucose ≥ 7 mmol/L or glucose levels 120 min after OGTT ≥ 11.1 mmol/L or HbA1c ≥ 48 mmol/mol, or use of anti-diabetic drugs). GMAs were considered at baseline evaluation in all patients, at diagnosis of acromegaly and before any specific treatment.

Our study complies with the STARD (standards for reporting diagnostic accuracy studies) criteria, and the Strengthening the Reporting of Observational Studies in Epidemiology (STROBE) statement and guideline [15].

The Ethics Committee of Padova University Hospital (Comitato Etico per la Sperimentazione Scientifica) approved the study (PP-PIT: Predictive and Prognostic factors in PITuitary Adenoma, AOP1782, CESC 4834/AO/20).

### OGTT

A standard (75 g) OGTT was performed in the morning, after an overnight fast. An indwelling catheter was inserted in a forearm vein, and the subject remained fast, supine, and resting throughout the test. Blood samples were collected right before, at 30, 60, 90, 120, 150, and 180 min after oral glucose intake.

GH was measured with Immulite 2000; analytical measurement range: 0.05 to 40 μg/L (0.15–120 mIU/L), metrological traceability: WHO NIBSC 2nd IS 98/574, with an intralaboratory coefficient of variation < 4%—low (3.28 μg/L), mid (8.25 μg/L) and high (13.00 μg/L) levels. IGF-1 levels were measured with LIAISON^®^ IGF-I (REF 313231 Diasorin), referenced to the 1st WHO International Standard for Insulin-like Growth Factor-I NIBSC code: 02/254, limit of detection 3 μg/L, limit of quantification 10 μg/L, assay range up to 1500 μg/L, intralaboratory imprecision < 5%. The levels within the normal range of IGF-1 for age and sex, correspond to the values specified by the manufacturer.

GH^basal^ was considered the fasting GH measured immediately before glucose load. Glucose-induced GH response was reported as the peak-to-basal GH ratio based on the OGTT (OGTT ratio). An OGTT ratio ≥ 120% achieved at last 90 min after glucose load was used to differentiate between subjects with (GH-Par) and without (GH-NPar) a paradoxical response of GH [9, 13]. Considering that “physiological” fluctuations can easily lead to a GH ratio ≥ 120% in the presence of low GH^basal^ levels (i.e., < 1 µg/L), we classified cases as paradoxical only when surpassing this threshold was associated with a GH^zenit^ exceeding 1 µg/L and with an absolute increase of at least 0.6 µg/L. For this reason, seven cases from the non-acro^↑IGF−1^ group and three cases from the non-acro group, despite a GH ratio ≥ 120%, were classified as GH-NPar.

### Statistical analysis

Continuous data were reported as mean and standard error; proportions and rates were calculated for categorical data. Groups were compared with the chi-square test for categorical variables (the raw *P* values were adjusted with the Bonferroni method to for multiple comparisons), and with the unpaired Student’s T test or Mann–Whitney Test for quantitative variables (when appropriate, after assessing the normality of distribution using the Kolmogorov–Smirnov Z test). We calculated the area under the curve (AUC) of GH, glucose, and insulin at the different OGTT time points with respect to the ground, according to the trapezoidal formula. The SPSS 24 software package for Windows (SPSS, Inc., Chicago, IL, USA) was used to manage the database and perform the statistical analysis. The significance level was set at *P* < 0.05 for all tests. All data analyzed during this study are included in the data repositories of the University of Padova—Research Data UniPD [17].

## Results

Demographic, clinical, and biochemical features of the cohort, stratified by the selection criteria (i.e., diagnosis of acromegaly, non-acro^↑IGF−1^, and non-acro), are presented in Table 1. As expected, the basal and post-OGTT GH and IGF-1 levels were lower in the absence of acromegaly. By definition, in the non-acro^↑IGF−1^ group, the median IGF-1 levels were higher than the non-acro group and above the age and gender-adjusted upper limit of normality (ULN, 1.33 ± 0.04). Prevalence of hypertension, GMA, and glucose or insulin levels (as well as HOMA-IR), was higher in acromegalic patients.

**Table 1:** Endocrine and clinical data in the three groups

	Acromegaly (n = 60)	Non-acro^↑IGF−1^ (n = 116)	Non-acro (n = 55)
Age (years)	52.1 (1.9)	44.5 (1.6)^a^	45.2 (2.2)^b^
Gender F/M (% F)	39/21 (65%)	72/44 (62%)	35/20 (64%)
GH^basal^ (µg/L)	16.19 (3.17)	2.71 (0.38)^a^	2.5 (0.5)^a^
GH^nadir^ (µg/L)	9.86 (1.27)	0.12 (0.05)^a^	0.1 (0.05)^a^
GH^AUC^	2930 (508)	182 (20)^a^	156 (22)^a^
GH^zenit^ (µg/L)	22.96 (3.99)	3.01 (0.33)^a^	2.59 (0.38)^a^
IGF-1 ULN	2.9 (0.15)	1.33 (0.04)^a^	0.75 (0.03)^a,c^
Glucose^basal^ (mmol/L)	5.5 (0.1)	5.2 (0.1)^b^	5.1 (0.1)^b^
Glucose^zenit^ (mmol/L)	10.8 (0.3)	9.5 (0.3)^a^	8.8 (0.4)^a^
Glucose^AUC^	1440 (40)	1287 (38)^b^	1208 (44)^a^
Insulin^basal^ (mU/L)	19 (3)	11 (2)^a^	8 (1)^a^
Insulin^zenit^ (mU/L)	185 (15)	127 (10)^a^	101 (10)^a^
Insulin^AUC^	19,862 (1774)	14,389 (1158)^a^	10,994 (1052)
HOMA-IR	4.84 (0.68)	2.6 (0.29)^a^	1.81 (0.23)^a^
HbA1c (mmol/mol)	39 (1)	38 (1)	36 (1)^a^
GMA normal/IFG/IGF/DM	7/16/9/19	8/22/10/48	5/6/3/32^b^
HT yes/no (% HT)	19/37 (34%)	22/94 (19%)^b^	7/48 (13%)^b^

Data are presented as mean and standard error (in brackets) or absolute number and percentage

*Non-acro*^*↑IGF−1*^ patients without acromegaly (GH^nadir^ < 0.4 µg/L) and increased IGF-1 level, *F* female, *M* male, *AUC* area under the curve, *ULN* upper limit of normality (gender and age), *nadir* the lowest value in the curve after basal, *zenit* the highest value in the curve after basal, *HOMA-IR* homeostasis model assessment-insulin resistance, *GMA* glucose metabolism abnormalities, *IFG* impaired fasting glucose, *IGT* impaired glucose tolerance, *DM* and diabetes mellitus, *HT* hypertension

^a^p < 0.001 vs acromegaly

^b^p < 0.05 vs acromegaly

^c^p < 0.001 vs non-acro^↑IGF−1^

**Fig. 1:**
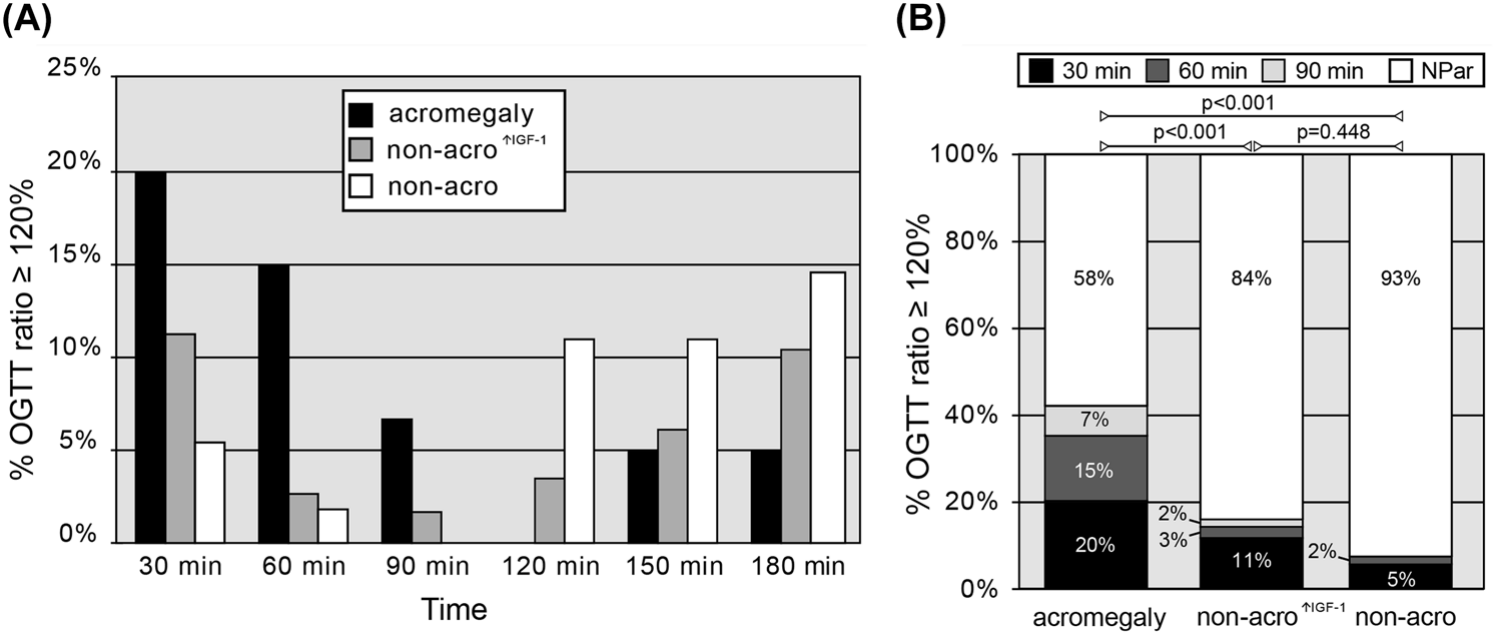
GH nadir above the 120% of basal level across the study populations. In **A**, we reported the frequency of patients with at least one GH ≥ 120% over basal levels, sorted by time of GH^zenit^. In **B** we depicted the frequency of GH^zenit^ ≥ 120% in the 3 populations considered: 30-min GH^zenit^ in black, 60-min GH^zenit^ in dark gray, 90-min GH^zenit^ in gray, late GH rise or GH suppression in white

To assess the extent of the paradoxical response of GH to oral glucose load among groups and distinguish it beyond any doubt from GH rebounds, the three groups were subdivided according to the presence of a GH ratio ≥ 120% during OGTT. As depicted in Fig. 1A, after 120 min from baseline, a similar proportion of subjects in all three groups show a GH ratio of ≥ 120%. In accordance with a previous hypothesis [14], the delayed increase in GH levels in these cases is probably due to a GH recovery to higher-than-baseline levels (i.e., GH rebound) after the rapid decrease brought about by hyperglycemia, rather than an authentic paradoxical increase. In other words, the effective peak of glucose-induced GH is observed no later than 90 min after glucose intake in most, if not all, patients. This prompted us to refine our definition of GH-Par, which now encompasses both the OGTT ratio (≥ 120%) and the timing of the peak (no later than 90 min after glucose load).

Based on these assumptions, we evaluated all of our cases and recorded the magnitude (in term of percentual increase above basal levels) and timing of the GH peak (GH^zenit^) that first exceeds the threshold, if any. As shown in Fig. 1B, about 40% of acromegalic patients present a paradoxical increase of GH after OGTT, which is consistent with our previous observations [18]. In both non-acro^↑IGF−1^ and non-acro groups, the prevalence of GH-Par is relatively uncommon, as expected (~ 16% and ~ 7%, respectively), with GH^zenit^ occurring in all but two non-acro^↑IGF−1^ cases within 60 min after the glucose challenge. Hence, when considering the prevalence of paradoxical responses, the non-acro^↑IGF−1^ group falls somewhere between acromegaly and the non-acro group.

As reported in Table 2 and consistent with previous reports, differences between GH-Par and GH-NPar within this group were noted [6, 7, 9, 10]. However, in some instances, such as the age at diagnosis and IGF-1 ULN, likely due to a limited sample size, statistical significance was not reached. Significant differences between GH-Par and GH-NPar subgroups emerges also within the non-acro^↑IGF−1^ group. The former, indeed, underwent the OGTT test at a younger age (34.4 *vs* 46.4 years, p = 0.006), which contrasts with the observations made in patients with acromegaly. Furthermore, in paradoxical cases, all GH-related parameters (i.e., GH^nadir^, GH^AUC^, and GH^zenit^) showed higher values, except for GH^basal^. On the other hand, non-acro^↑IGF−1^ GH-Par patients have poorer glycemic control and higher basal glucose levels compared to those without paradoxical response and present a reduced prevalence of pituitary adenomas (13% *vs* 39%, p = 0.049). In the non-acro group, variations in the prevalence of pituitary adenomas and in the age at which subjects underwent the OGTT between Par and NPar can also be observed. However, it is worth noting that these differences may be primarily influenced by the unequal group sizes, resulting in a tendency rather than a definitive difference. Finally, within the GH-Par non-acro group, the non-stimulated GH^basal^ levels are significantly lower compared to the non paradoxical counterpart. As for GH^zenit^, GH^nadir^, and GH^AUC^, the observed differences align with the anticipated outcomes according to the defined criteria.

**Table 2:** Endocrine and clinical data in GH-Par (paradoxical GH response to OGTT, OGTT ratio: ≥ 120%, at latest 90 min after the glucose load) and GH-NPar (non-paradoxical GH response to OGTT)

	Acromegaly (n = 60)			Non-acro^↑IGF−1^ (n = 116)			Non-acro (n = 55)		
	GH-Par (n = 25)	GH-NPar (n = 35)	p	GH-Par (n = 18)	GH-NPar (n = 98)	p	GH-Par (n = 4)	GH-NPar (n = 51)	p
Age (years)	56.7 (3.2)	48.9 (2.4)	0.051	34.4 (3.6)	46.4 (1.8)	**0.006**	31.2 (10.7)	46.4 (2.2)	0.072
Gender F/M (% female)	17/8 (68%)	22/13 (63%)	0.786	10/8 (56%)	62/36 (63%)	0.536	3/1 (75%)	32/19 (63%)	0.624
GH^basal^ (µg/L)	12.1 (2.41)	19.12 (5.13)	0.278	1.51 (0.66)	2.92 (0.43)	0.08	0.13 (0.05)	2.66 (0.56)	**0.016**
GH^nadir^ (µg/L)	11.51 (1.95)	8.69 (1.67)	0.277	0.18 (0.02)	0.1 (0.01)	** < 0.001**	0.12 (0.03)	0.1 (0.01)	0.717
GH^AUC^ (µg/L)	3368 (736)	2616 (697)	0.47	382 (87)	145 (15)	** < 0.001**	284 (44)	145 (23)	**0.025**
GH^zenit^ up to 90 min (µg/L)	26.01 (5.81)	17.94 (5.3)	0.315	5.67 (1.29)	1.25 (0.17)	** < 0.001**	3.65 (1.56)	0.96 (0.24)	**0.006**
IGF-1 ULN	2.97 (0.27)	2.84 (0.17)	0.696	1.29 (0.09)	1.34 (0.04)	0.645	0.8 (0.05)	0.74 (0.03)	0.409
Glucose^basal^ (mmol/L)	5.75 (0.16)	5.34 (0.13)	0.053	4.84 (0.11)	5.25 (0.09)	**0.008**	6.27 (1.92)	5 (0.1)	0.58
Glucose^zenit^ (mmol/L)	11.56 (0.57)	10.28 (0.3)	**0.038**	8.58 (0.45)	9.63 (0.3)	0.064	7.57 (0.95)	8.94 (0.34)	0.28
Glucose^AUC^	1535 (72)	1372 (42)	**0.044**	1186 (64)	1306 (44)	0.132	1100 (136)	1216 (46)	0.518
Insulin^basal^ (mU/L)	20 (5)	19 (3)	0.846	11 (2)	11 (1)	0.951	10 (3)	8 (1)	0.415
Insulin^zenit^ (mU/L)	185 (25)	185 (18)	0.993	127 (29)	127 (10)	0.995	110 (18)	100 (10)	0.663
Insulin^AUC^	19,951 (3301)	19,803 (2026)	0.968	1429 (3188)	14,405 (1251)	0.975	12,265 (1577)	10,906 (1121)	0.517
HOMA-IR	5.19 (1.33)	4.61 (0.71)	0.674	2.48 (0.49)	2.62 (0.33)	0.807	2.52 (0.34)	1.76 (0.25)	0.136
HbA1c (mmol/mol)	39 (1)	38 (1)	0.558	33 (1)	38 (1)	**0.005**	33 (1)	36 (1)	0.306
GMA normal/IFG/IGF/DM	3/3/6/6	13/6/10/1	0.079	10/0/4/0	38/10/18/8	0.225	2/0/0/1	30/3/6/4	0.548
HT yes/no (% HT)	9/15 (38%)	10/22 (31%)	0.625	2/16 (11%)	20/78 (20%)	0.355	0/4 (0%)	7/44 (14%)	0.428
Pituitary adenoma yes/no	25/0 (100%)	34/1 (97%)	0.394	2/13 (13%)	34/54 (39%)	**0.049**	1/3 (25%)	18/23 (43%)	0.079

Data are presented as mean and standard error (in brackets) or absolute number and percentage. The p value reported in the last column of each group referred to the comparison between GH-Par and GH-NPar patients within this group. In case of statistical significance, p values are displayed in bold. Pituitary adenoma: number are referred in patients with a pituitary MR available

*Non-acro*^*↑IGF−1*^ patients without acromegaly (GH^nadir^ < 0.4 µg/L) and increased IGF-1 level, *F* female, *M* male, *AUC* area under the curve, *ULN* upper limit of normality (gender and age), *nadir* the lowest value in the curve after basal, *zenit* the highest value in the curve after basal, *OGTT ratio* the ratio GH^zenit^ to GH^basal^, *HOMA-IR* homeostasis model assessment-insulin resistance, *GMA* glucose metabolism abnormalities, *IFG* impaired fasting glucose, *IGT* impaired glucose tolerance, *DM* and diabetes mellitus, *HT* hypertension

**Fig. 2:**
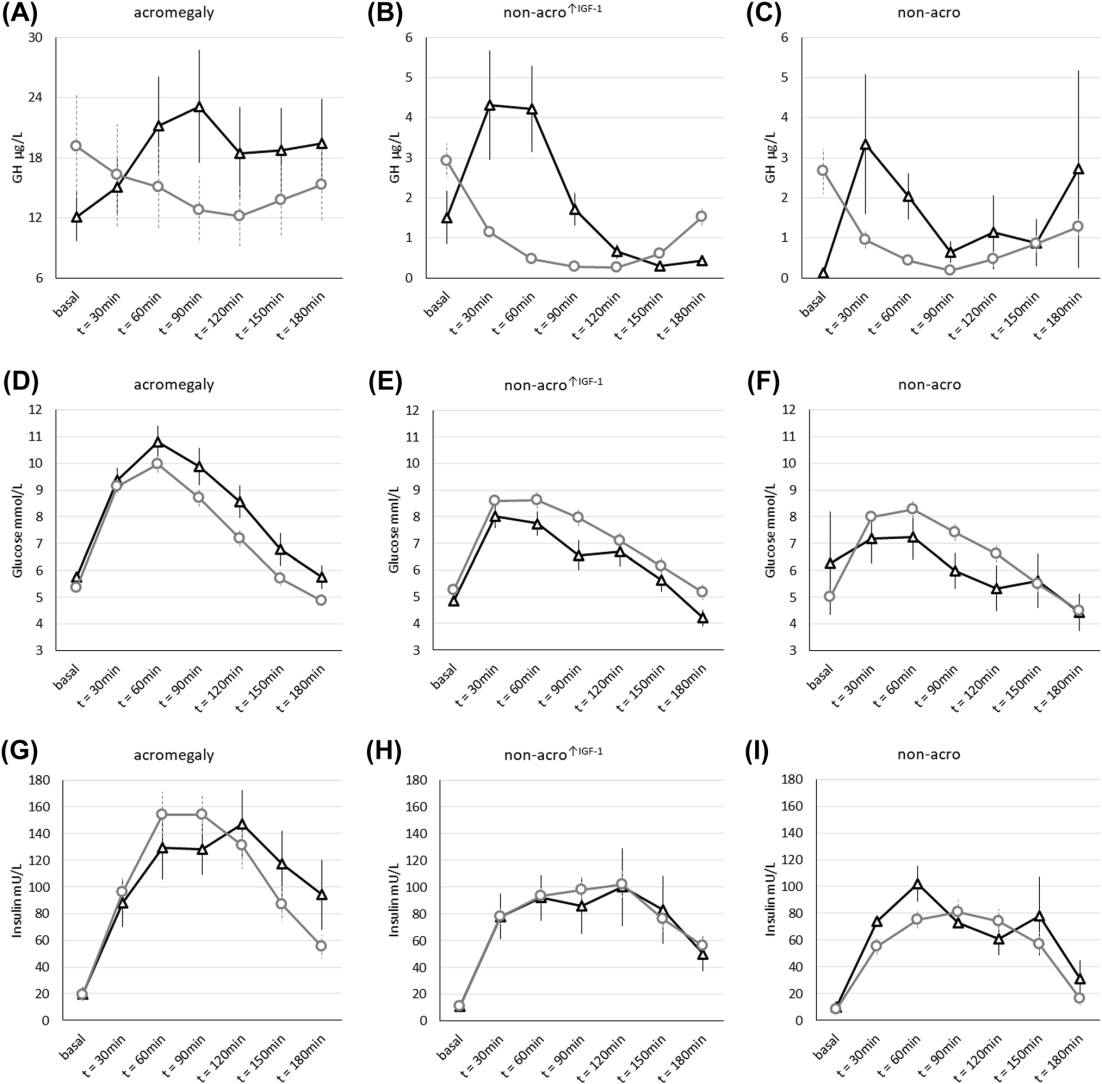
Levels of GH (**a**–**c**), glucose (**d**–**f**), and insulin (**g**–**i**) during OGTT in patients with acromegaly, non-acro^↑IGF−1^ and non-acro. Curves are divided in patients with paradoxical GH response to OGTT (GH-Par, defined as OGTT ratio ≥ 1.2 at latest 90 min after glucose load, black with triangles) or without paradoxical GH response (GH-NPar, grey with circles). Data are given as means and standard errors

As depicted in Fig. 2 (panels a-c), the shape of the GH response curve during the OGTT of GH-Par subjects was very similar across the three groups: an early increase of GH after a glucose challenge was followed by a partial (in acromegaly) or complete GH suppression (in non-acro^↑IGF−1^ and non-acro). Conversely, the kinetics of the glycemic response curve associated with GH-Par differed significantly (Fig. 2d–f). In acromegaly patients, the glucose peak was higher and the drop in glucose levels was slower compared to the other two groups, resulting in higher glucose in the second half of the curve. This leads to a 20% higher glucose AUC despite similar insulin secretion (Table 2; Fig. 2g–i). Increased glucose exposure was associated with a higher prevalence of diabetes in acromegaly GH-Par, with a similar (considering concentration) but late (1 h) insulin peak in respect to GH-NPar (Fig. 2g).

## Discussion

Failure to suppress GH during OGTT is the cornerstone in the diagnosis of acromegaly in patients with increased IGF-1 levels [3, 19]. In recent studies, a prognostic potential of GH levels after OGTT has also been highlighted [6–9]. Paradoxical GH response to a glucose challenge is more common in patients with a milder tumoral phenotype, including a better response to medical therapy [6–9]. In addition, GH-Par exhibited unique MRI features and was associated with significant differences in glucose metabolism parameters [6, 7], as observed in patients with Cushing’s Syndrome [20]. An ectopic GIP/GIPR axis in tumor somatotroph cells causes ~ 80% of cases of the paradoxical responses of GH [10–13]. While the diagnosis of acromegaly is based on well-established criteria, there is no consensus on how to define a paradoxical GH response after OGTT. Different studies use varying standards/inclusion criteria, concerning the magnitude of the increase—ranging from 20 to 100%—and/or timing of peak measurement—i.e., within either 90 min or 120 min. The matter is even more complicated as the paradoxical GH response to OGTT is not restricted to acromegaly but may manifest in other physiological and pathological conditions as well [14]. In this study, we evaluated the GH profiles over 180 min OGTT in three groups of subjects, one consisting of acromegalic patients. We found that in the latest time points after glucose load (≥ 120 min) the frequency of GH peaks exceeding the 120% threshold is quite similar among the three groups. This confirms the previous hypothesis of appropriateness to limit to the first 90 min to define a paradoxical increase of GH in acromegaly [14]. Beyond this time point, indeed, the fluctuations my reflect a GH rebound, resulting from a decrease in the somatostatinergic tone, a consequent increase in GHRH and the release of pituitary GH stores [21]. Considering the current and past research on molecular data [11], in acromegaly it may be appropriate to define a paradoxical increase in response to an oral glucose challenge as a 20% increase occurring within the first 90 min. In other conditions, where the mechanisms underlying the lack of glucose suppression on GH remain unclear, we consider it reasonable to employ the same criterion only when basal GH levels are adequately high. Conversely, in cases of low GH basal levels, taking into account both the absolute increase and the GH^zenith^ value—as we did in this study—appears equally reasonable.

According to this new definition of paradoxical response, we observed that the prevalence of GH-Par in the non-acro^↑IGF−1^ group falls between the acromegalic patients and non-acro. The prevalence found in this latter is consistent with the literature [15], despite using different selection criteria. The GH-Par in non-acro is an immediate event, achieving GH zenit early after glucose load (within 30 min). This consistency, which likely reflects the exclusion from our cohort of conditions already associated with GH-Par, suggests that a certain part of the general population may physiologically experience these responses. The exact underlying mechanisms, however, are yet to be determined.

Of particular interest is the frequency of GH-Par that emerges from this study in the non-acro^↑IGF−1^ group, which includes subjects with high IGF-1 levels and without acromegaly (excluded with the most sensitive threshold of GH^nadir^ < 0.4 µg/L after OGTT). The frequency of GH-Par in non-acro^↑IGF−1^ is intermediate between acromegaly and non-acro. While it may be difficult to interpret this similarity based on the data from this study alone, we can certainly speculate about it. Recently, we have confirmed that the aberrant expression of GIPR is an early event in the neoplastic transformation in medullary thyroid cancer [22, 23], which present a high tumor-to-normal tissue ratio (T/N ratio) for GIPR, like most neuroendocrine tumours [23]. On the other hand, the neoplastic transformation in primary bilateral macronodular adrenal hyperplasia (PBMAH) involves the ectopic expression of GIPR, which is necessary and sufficient for this condition to occur [24]. Since GIPR mediates the paradoxical response in more than 80% of somatotropinomas [10, 11] it can be hypothesized that, at least in some cases of the non-acro^↑IGF−1^ group, the GH-Par may represent an early indicator of acromegaly. In support of this hypothesis, high circulating GIP levels in acromegaly [25] may be due to increased IGF-1 levels, which induce GIP synthesis in the intestinal neuroendocrine cell line STC-1 [11]. This suggests the development of a possible vicious cycle involving GH/IGF-1/GIP and their receptors in the liver, duodenum, and somatotroph tumor cells in the early stages of the disease. In this phase, this axis might be triggered in response to meal but then become chronic over time [18]. Our observations of a younger age and lower incidence of pituitary adenomas in individuals with GH-Par but without acromegaly, as compared to acromegalic patients, could support this hypothesis.

In such a scenario, a long-term observational study could help determine the incidence of new-onset acromegaly in non-acro^↑IGF−1^ GH-Par subjects and elevated IGF-1 levels. In addition, some models may offer valuable insights for testing this hypothesis, as the positron emission tomography with GIP-based radioligand [26], or the development of a transgenic model expressing GIPR at the pituitary level in a conditional and inducible manner. The present study's findings undoubtedly strengthen the case for developing such a model.

In our cohort, acromegalic subjects with GH-Par present a slower decline in glucose levels and a higher glucose peak compared to those with GH-NPar. This results in a higher glucose level in the latter part of the curve, thus leading to a 10% increase in glucose exposure after meal (in terms of AUC). Moreover, these patients are characterized by delayed insulin secretion, with an insulin peak occurring about 60 min after the non-paradoxical acromegalic patients. Considering GMA, we observed a trend towards a higher prevalence of diabetes associated with GH-Par acromegaly (~ 24% vs ~ 3%), which agrees with previous data [6, 7]. The use of novel somatostatin analogs, such as Pasireotide [27], can impair glucose metabolism: the prediction of Pasireotide's efficacy and adverse effects is of particular interest in GH-Par patients. Recent reports suggest that Pasireotide-induced diabetes is mediated by a rapid impairment of insulin secretion without different insulin sensitivity, especially in older acromegalic patients with poor baseline glycemic control [28]. This data indicates that GH-Par acromegalic patients have a high risk of developing diabetes after Pasireotide treatment, as they tend to be older with elevated fasting glucose levels. Moreover, the increased glucose exposure (in terms of AUC) in acromegaly GH-Par may represent another paradox. As increased glucose levels are required to suppress GH, the higher glucose means higher GH suppression. On the contrary, we observe the peak of GH levels (they are acromegalic GH-Par by definition) at the 60–90 min time point of the curve, shortly after the glucose peak at 30–60 min. To verify the hypothesis of diabetes prevalence in GH-Par, a larger cohort of patients in a prospective study is necessary, and animal models may provide insights into the connection between GH-Par, glucose, and insulin.

Despite its strengths, our work presents some limitations too. It is an observational study, without intervention in unselected patients with suspected acromegaly. In addition, we observed a high prevalence of pituitary adenomas in the control groups, higher than that in the normal population. This is a direct consequence of our clinical practice. Patients were examined at our pituitary referral outpatient clinic, where pituitary imaging is routinely performed before the first visit, as suggested by the referring physician or general practitioner.

## Conclusion

Our paper provides new evidence that allows for a revision of the definition of GH-Par. Specifically, the revised definition associates the magnitude of the increase with the timing of the peak. Here we also described for the first time a paradoxical GH increase after OGTT in a subset of non-acromegalic subjects with high IGF-1 levels, that support the hypothesis that an aberrant GIPR expression could be an early event in the GH-sec PA tumorigenesis. Further studies are needed to establish the connection between oral food intake, GIP/GIPR axis, and the development of pituitary adenoma.

## Data Availability

All data generated or analyzed during this study are included in this published article or the data repositories listed in the references.
